# The impact of highly active antiretroviral therapy on prevalence and incidence of cervical human papillomavirus infections in HIV-positive adolescents

**DOI:** 10.1186/1471-2334-10-295

**Published:** 2010-10-14

**Authors:** Sadeep Shrestha, Staci L Sudenga, Jennifer S Smith, Laura H Bachmann, Craig M Wilson, Mirjam C Kempf

**Affiliations:** 1 Department of Epidemiology, School of Public Health, University of Alabama at Birmingham, Birmingham, Alabama, USA; 2 Department of Epidemiology, Gillings School of Global Public Health, University of North Carolina, Chapel Hill, North Carolina, USA; 3 WG (Bill) Hefner Medical Center, Salisbury, North Carolina, USA; 4 Wake Forest University Health Sciences, Winston-Salem, North Carolina, USA

## Abstract

**Background:**

The implementation of highly active antiretroviral therapy (HAART) among HIV-positive patients results in immune reconstitution, slower progression of HIV disease, and a decrease in the occurrence of opportunistic infections. However, the impact of HAART on cervical human papillomavirus (HPV) infection, clearance, and persistence in high-risk adolescents remains controversial.

**Methods:**

HIV-positive and high-risk HIV-negative female adolescents were enrolled in the Reaching for Excellence in Adolescent Care and Health (REACH) longitudinal cohort study. At each semi-annual clinical visit, cervical lavage samples were tested for 30 HPV types. Type-specific and carcinogenic risk-specific HPV prevalence and incidence were compared in 373 eligible participants: 146 HIV-negative female adolescents with a median follow-up of 721.5 [IQR: 483-1301] days and 227 HIV-positive female adolescents. Of the 227 HIV-positive participants, a fixed set (n = 100) were examined both before and after HAART initiation; 70 were examined only before HAART initiation; and 57 were examined only after HAART initiation, with overall median follow-up of 271 [IQR: 86.5-473] and 427.25 [IQR: 200-871] days respectively for before and after HAART initiation.

**Results:**

Of the 373 eligible participants, 262 (70%) were infected with at least one type of HPV at baseline, and 78 of the remaining 111 (70%) became infected with at least one type of HPV by the end of the study. Overall, the incidence and prevalence of HPV types 58, 53/66, 68/70, and 31/33/35 were much higher than the established carcinogenic and HPV vaccine types 16 and 18, especially in HIV-positive females both before and after HAART initiation. Baseline prevalence for individual high-risk HPV types ranged, depending on type, from 0.7-10%, 1-17%, and 1-18% in the HIV-negative group, the HIV-positive before HAART initiation group, and the HIV-positive after HAART initiation group, respectively. Likewise, the incidence ranged, depending on HPV type, from 0.64-9.83 cases/100 PY, 3.00-12.80 cases/100 PY, and 1.49-17.05 cases/100 PY in the three groups, respectively. The patterns of each HPV type infection, clearance, and persistence did not differ considerably before or after the introduction of HAART and were clearly independent of CD4^+ ^change within the short post-HAART follow-up period.

**Conclusions:**

HAART did not immediately affect the incidence of type-specific HPV infections within a short-period follow-up; however, future studies are warranted in larger populations to evaluate HAART's impact over longer periods.

## Background

Human papillomavirus (HPV) infection is the most prevalent sexually transmitted infection (STI) in the United States (U.S.) and the world [1,2]. In the NHANES survey, the overall HPV prevalence was 24.5% (95% CI, 19.6%-30.5%) among females aged 14-19 years [3]. Of the estimated 20 million infected persons in the U.S., about half are 15-24 years old; another 6.2 million new cases (4.6 million aged 15-24) are diagnosed annually [4]. Furthermore, an estimated 80% of all sexually active females will have been exposed to HPV by age 50 [5]. The medical care cost for HPV-related conditions (including abnormal Pap smears and treatment for cervical neoplasia and cancer) is over $3.5 billion per year--greater than for all other common STIs except HIV/AIDS [6]. Although most infected individuals (70-90%) naturally clear an HPV infection within 12-24 months, the virus persists in a subset of infected hosts [7-9]. An infection that has persisted for more than four years has only a small chance of remission [10]. Persistent HPV infection, along with environmental and genetic factors, predisposes individuals to the development of high-grade cervical intraepithelial neoplasia (CIN) [11] and, in some cases, to subsequent progression to cancer [12]. Other clinical manifestations of HPV infection include anogenital warts and recurrent respiratory papillomatosis, as well as anal, vulvar, vaginal, penile, and head and neck cancers.

HIV-negative females effectively clear cervical HPV infections 4-10 times more frequently than HIV-positive females, with the lowest rate of clearance among severely immunocompromised females with CD4^+ ^T-cell (CD4^+^) counts <200 (cells/mm^3^) [13-15]. A previous study among participants of the Reaching for Excellence in Adolescent Care and Health (REACH) cohort suggested that the frequency of HPV persistence varied inversely with CD4^+ ^count [16]. Likewise, other studies have found higher HPV prevalence and incidence of oncogenic HPV types in HIV patients, especially those with lower CD4^+ ^counts, compared to HIV-negative individuals [13,17,18]. These data suggest that the level of CD4^+ ^is important in the pathogenesis of HPV infection in HIV patients.

The cervical cancer incidence among HIV-infected females in the U.S. is higher (86.5 cases per 100,000 person-years [PY]) since the introduction of HAART (1996-2002) than it was in 1990-1996 (64.2 cases per 100,000 PY), although the difference is not statistically significant (relative risk [RR] = 1.41, 95% CI = 0.81 to 2.46) [19]. This could be attributed to the fact that, in the HAART era, most HIV-positive females live longer and thus the cumulative prevalence of cervical cancer could be higher. The widespread use of HAART, resulting in immune reconstitution for HIV-positive patients, has led to a decrease in the incidence of opportunistic infections and HIV-related malignancies such as Kaposi's sarcoma and non-Hodgkins lymphoma. However, no association between CD4^+ ^count and cervical cancer incidence was observed in either period in the same study [19]. Clearly, females with AIDS have an increased risk of cervical cancer, but whether this risk is related to immunosuppression or simply reflects a higher risk of acquiring human papillomavirus in the same population is unclear.

Studies of the impact of HAART on the natural history of HPV infection and HPV-related diseases, however, are limited and inconsistent [20]. While some studies show clear reductions in progression of lesions or disease [21-25], others have shown negative or no effects [26-30]. Most studies examining the effect of HAART on HPV-related outcomes have focused on cervical precursor lesions or cancer, but not on HPV clearance or persistence. With the increase of CD4^+ ^T-cells and the decrease in HIV viral load through HAART therapy, immune reconstitution as a result of HAART may be associated with the clearance of HPV infection. In this report, the immediate effect of HAART on the incidence, persistence, and clearance of type-specific HPV among HIV-positive female adolescents is examined.

## Methods

### Study population

Participants in this analysis were HIV-positive and HIV-negative female adolescents enrolled in the Reaching for Excellence in Adolescent Care and Health (REACH) study. The study design and methods for quarterly follow-up, HIV-1 testing, immunophenotyping of CD4^+ ^counts, and collection of biological specimens along with demographic, risk behavior, and other clinical data for this cohort have been previously described in detail [31,32]. Briefly, adolescents aged 12-19 years who acquired HIV-1 through sexual activity or injection drug use (perinatal transmission or blood product contamination were excluded) and comparable high-risk seronegatives were recruited between February 1996 and November 2000 and followed up with until December 2000 in a longitudinal study at 15 clinical sites in the U.S. to investigate the natural history of HIV [33]. Of the 548 adolescents enrolled in the REACH study, 355 were HIV-seropositive (71% African-Americans, 75% females, 91% with CD4^+ ^≥200 at baseline), and two sero-converted and were excluded from the study. We included follow-up data from all HIV-negative and HIV-positive female adolescents off HAART (HAART-naïve throughout the study or until initiation) and follow-up data from all HIV-positive female adolescents initiating HAART during the REACH study.

For the case-crossover sub-study, we included only HIV-positive female adolescents who were HAART-naïve at enrollment but initiated HAART during the course of the study, providing adequate follow-up periods before and after the introduction of HAART (referred to as before HAART initiation and after HAART initiation). This allows for internal controlling of potential confounders. Therefore, those who self-reported previous initiation of HAART or had chart documentation of HAART initiation prior to enrolling in the REACH cohort, as well as those who did not initiate HAART during the study, were excluded from this sub-study sensitivity analysis. All the participants provided written informed consent in the parent study, and the UAB Institutional Review Board approved this sub-study.

### HAART Treatment

Participants who were prescribed HAART, consistent with the Public Health Service Guidelines for the Use of Antiretroviral Agents in HIV-Infected Adults and Adolescents [34], were included in the analysis. At the time of the study visit, HAART was defined as a combination of two nucleoside reverse transcriptase inhibitors and either a protease inhibitor or a non-nucleoside reverse transcriptase inhibitor, or a zidovudine/lamivudine combination regimen plus another antiretroviral drug. HPV data from the last study visit before the initiation of HAART were used as baseline values for comparison to after HAART initiation values in the subset group. Data on antiretroviral therapy were obtained through interview and chart review for current prescriptions, and adherence data were obtained through interviews as previously described [35]. Participants were censored at the end of the study, or at the last available visit or the first visit that they reported not adhering to the HAART regimen.

### HPV DNA Detection

At enrollment and every six months thereafter, cervical lavage samples were tested for the presence of HPV. In brief, viral DNA fragments from the samples were amplified by use of consensus primers MY09/11 and HMB01 and hybridized with a consensus probe and 30 different HPV strain-specific probes (some probes were, however, specific for more than one HPV type) by use of a chemiluminescent dot-blot format [36]. PCR-based HPV data were classified as follows: negative; positive for the specific types; or "positive, type unknown" if the sample was positive for the generic probe but not for a specific HPV type. PCR amplification of the human β-globin gene segment was used as an internal control for DNA quality, and samples negative in this assay were excluded from analyses. There were 21 β-globin negative observations among HIV-negative female adolescents, of whom four were HPV-positive; nine among HIV-positive female adolescents who were never on HAART, of whom two were HPV-positive; and 38 during HAART, of whom three were HPV-positive. For analytic purposes, HPV types also were categorized according to risk groups [37,38] and by vaccine coverage: i) high-risk carcinogenic (16, 18, 31, 33, 35, 39, 45, 51, 52, 56, 58, 59, and 68); ii) possible carcinogenic (26, 53, 66, 67, and 69); iii) low-risk (2, 6, 11, 13, 32, 40, 42, 44, 54, 55, 57, 62, and 72); and iv) bivalent vaccine type (16 and 18).

### Before and After HAART Initiation Period

The before HAART initiation follow-up duration was estimated from the date of enrollment to the last visit before starting HAART. After HAART initiation follow-up included the time from the start date of HAART to either the end of the REACH study, the last visit before HAART was stopped, the visit at which the patient self-reported not adhering to HAART, or when the patient was lost to follow-up--whichever was applicable.

### Statistical Analysis

Baseline prevalence and incidence of type-specific HPV infections were estimated and compared among different groups within the REACH cohort: 1) before HAART initiation period only; 2) after HAART initiation period only; 3) HIV-negatives; and 4) a case-crossover subset with both before and after HAART initiation periods. Cumulative prevalence was defined as the percent of female adolescents with an HPV infection detected at either the baseline or at one of the follow-up visits. The type-specific HPV incidence per 100 PY, along with 95% confidence intervals (95% CI), were estimated before and after HAART initiation, and statistical differences were reported with p-values using chi-square tests. To contribute person-time to the before HAART initiation incidence analyses, participants were required to be HPV-negative for a specific HPV strain at baseline, and for after HAART initiation incidence analyses, participants had to be HPV-negative for a specific HPV strain from the before HAART initiation period until the start of HAART. Since exact infection dates are not known, the midpoint between the last HPV-negative visit and the first HPV-positive visit date was used as the estimated infection incident date for calculation of person-time at risk. Time elapsed following the HPV incidence date was not included in estimates of person-time at risk. However, for participants testing negative for a specific HPV type during the before and after HAART initiation periods, person-time was estimated from the beginning through the end of the study in the two periods, respectively. Participants co-infected with different HPV types were analyzed separately for each HPV type. The prevalence and incidence by HIV status and before and after HAART initiation periods were compared.

We also compared the CD4^+ ^counts of the before HAART initiation infection with after HAART initiation clearance (the average CD4^+ ^counts from the last HPV-positive before HAART initiation visit and the first of the two consecutive HPV-negative visits after infection in the after HAART initiation for all HPV types), persistence (the average CD4+ during all infected visits before and after HAART initiation, and for new infection (average CD4^+ ^counts from the last HPV-negative visit before HAART initiation and the first HPV-positive visit after HAART initiation).

## Results

There were a total of 373 eligible female adolescent participants (146 HIV-negative and 227 HIV-positive) in the REACH cohort, of whom 262 (70%) were infected with at least one HPV type at baseline. Of the remaining 111 not infected with HPV at baseline, 78 (70%) became infected with at least one type of HPV by the end of the study. Of the HIV-positives, 192 were HAART-naïve at enrollment but initiated HAART while participating in the REACH study; 62 started HAART on the first visit and lacked before HAART initiation follow-up; 22 had missing dates for the initiation of HAART; and seven lacked adequate follow-up data after HAART initiation. The prevalence and incidence estimates were based on 146 HIV-negative females, 170 for the before HAART initiation period (100 of whom initiated HAART during the study and were censored up until that date, and another 70 who were never on HAART), and 157 for the after HAART initiation period (57 of whom initiated HAART immediately after enrollment and therefore contributed no before HAART initiation data, and the 100 in the case-crossover subset with at least two follow-up visits before and after HAART with HPV data). There were a total of 16 reappearances of type-specific HPV (after two consecutive negative visits) among 100 individuals during the study period. Three reappeared during pre-HAART, three during post-HAART, and 10 were infected pre-HAART, cleared post-HAART, but reappeared post-HAART. Although the numbers were small, there is no significant pattern of reappearance with or without HAART.

The demographic and baseline clinical characteristics of the subjects included in this sub-study and the entire REACH cohort did not differ significantly, as shown in Table 1. Additionally, self-reported numbers of sex partners in the past three months (39% vs. 34% with no partner, 36% vs. 40% with 1 partner, 19% vs. 22% with 2-4 partners, and 5% vs. 4% with ≥5 partners) and condom use in the last sexual encounter (62% vs.70% who said yes) were similar between the participants of this study and all REACH female participants [31]. The median follow-up time for HIV-negative participants was 721.5 days [Interquartile range (IQR) 483-1301] with 5 [IQR 3-7] visits, and for participants with before/after HAART initiation crossover study visits, the before HAART initiation follow-up time was 271 days [IQR 86.5-473] with 2 [IQR 1-3] visits, and after HAART initiation follow-up time was 427.25 days [IQR 200-871] with 2.5 [IQR 1-5] visits. In Table 1, CD4^+ ^count seems to be higher in participants before HAART initiation than after HAART initiation, but it is noteworthy that some of the sicker individuals (low CD4^+^) who were treated did not have before HAART initiation visits included in the study. However, among the cross-over set, there was an increase of CD4^+ ^after HAART initiation (mean increase from 482 to 560 and a median increase from 471 to 525 cells/mm^3 ^within a six-month period), indicating an overall immune reconstitution.

**Table 1:** Comparisons of demographics and baseline characteristics of participants stratified by HIV status and HAART time period in the REACH cohort

Variables	Participants				
	
	REACH Females (n = 411)	REACH HIV- (n = 146)	All Before HAART Initiation (n = 170)	All After HAART Initiation (n = 157)	Case-crossover with Both Before and After HAART Initiation (n = 100)
**Baseline Age**					
**Median [IQR]**	17 [16-18]	17 [16-18]	17 [16-18]	17 [16-18]	17 [16-18]
**Mean (std dev)**	16.25 (1.18)	16.59 (1.21)	17.37(1.41)	17.42 (1.36)	16.88(1.12)
					
**Race**					
**African-American**	308 (75%)	122 (84%)	132 (78%)	117 (75%)	72 (72%)
**European-American**	71 (17%)	20 (14%)	23 (14%)	22 (15%)	19 (19%)
**Other**	29 (7%)	4 (2%)	15 (8%)	16 (10%)	9 (9%)
					
**Lifetime Sexual Partner**					
**Median [IQR]**	6 [3-11]	5 [3-9]	6 [3-10]	7 [4-11]	6 [4-16]
					
**CD4**^**+ **^**Count (at Baseline)**					
**Median [IQR]**	614.0 [425-860]	825.5 [672-1016]	516.5 [389-730]	478.5 [344-620]	481 [370-616]
**Mean (std dev)**	649.8 (310.9)	858.5 (282.21)	557.3 (261.17)	486.4 (244.6)	498.1 (239.5)

As shown in Table 2, type-specific high-risk carcinogenic HPV baseline prevalence during the before HAART initiation period ranged from 1-17% and during the after HAART initiation from 1-18%, and amongst HIV-negatives, the prevalence ranged from 0.7-10%. HPV type 58 was the most prevalent at baseline among HIV-negatives (10%, 95%CI: 5-14%), while HPV 16 was most prevalent in the before HAART initiation period (10%, 95%CI: 11-22%) and HPV 53/66 in the after HAART initiation period (18%, 95%CI: 12-24%). However, type-specific high-risk carcinogenic HPV cumulative prevalence during the before HAART initiation period ranged from 6-29% and during the after-HAART initiation from 4-43%, and amongst HIV-negatives, the prevalence ranged from 2-26% with possible carcinogenic HPV type 53/66, and not HPV 16 or HPV 18, being the most prevalent in all the groups. Among the low-risk type, HPV 6/11/42/44 had the highest baseline and overall cumulative prevalence in all three groups. We note that in both cases of HPV 53/66 and 6/11/42/44, the detection probes are specific to more than one HPV and thus could result in the high frequency.

**Table 2:** Prevalence and incidence of individual HPV types among HIV-negative and HIV-positive adolescent women participating in the REACH study during the before and after HAART initiation period

	ALL Participants						Case-crossover with Both Before and After HAART Initiation			
		
	Prevalence [95% CI] (%)			Incidence [95% CI] (cases/100 person-years)			Prevalence [95% CI] (%)		Incidence [95% CI] (cases/100 person-years)	
	
HPV Types	**HIV**^- ^**n = 146**	**HIV**^**+ **^**Before HAART n = 170**	**HIV**^**+ **^**After HAART n = 157**	**HIV**^- ^**n = 146**	**HIV**^**+ **^**Before HAART n = 170**	**HIV**^**+ **^**After HAART n = 157**	Before HAART n = 100	After HAART n = 100	Before HAART n = 100	After HAART n = 100
**Carcinogenic**										
HPV 16^a, c^	6 (2-10)	17 (11-22)***	10(5-14)	6.83 (3.53-11.96)	6.54 (3.38-11.45)	6.67 (3.45-11.68)	20 (12-28)^§§^	7 (2-12)	4.12 (2.13-7.20)	4.64 (2.40-8.12)
HPV18^a, c^	8 (3-12)	8 (4-12)	6(2-9)	4.66 (2.41-8.15)	6.27 (3.24-10.97)	7.32 (3.78-12.81)	10 (4-16)	4 (0-8)	8.49 (4.39-14.86)	5.72 (2.96-10.02)
HPV39^a^	0.7 (0-2)	4(1-7)*	4 (1-8)^#^	1.93 (0.99-3.37)	3.00 (1.55-5.25)	3.06 (1.58-5.36)	7 (2-12)	3 (0-6)	4.73 (2.45-8.28)	1.50 (0.78-2.63)
HPV45^a^	2 (0-6)	5 (2-8)	4 (1-8)	3.92 (2.02-6.85)	4.30 (2.23-7.53)	4.66 (2.41-8.16)	5 (1-9)	5 (1-9)	4.55 (2.35-7.97)	3.91 (2.02-6.84)
HPV51^a^	3 (0-6)	2 (0-5)	3 (0-5)	4.86 (2.51-8.51)	4.67 (2.41-8.17)	4.48 (2.31-7.83)	1 (0-3)	2 (0-5)	4.42 (2.29-7.74)	3.68 (1.90-6.45)
HPV52^a^	3 (0-5)	10 (5-15)	9 (4-13)^#^	4.67 (2.41-8.17)	5.02 (2.59-8.78)	5.11 (2.64-8.95)	12 (6-18)^§^	4 (0-8)	7.26 (3.76-12.71)	3.17 (1.64-5.55)
HPV56^a^	2 (0-4)	8 (4-12)*	4 (1-7)	2.60 (1.34-4.56)	5.44 (2.81-9.53)	4.21 (2.18-7.37)	8 (3-13)^§^	1 (0-3)	7.14 (3.69-12.49)	5.98 (3.09-10.47)
HPV58^a^	10 (5-14)	16 (11-22)	10 (5-15)	7.36 (3.80-12.88)	7.71 (3.99-13.49)	7.04 (3.64-12.32)	20 (12-28)^§§§^	6 (1-11)	6.93 (3.58-12.13)	5.93 (3.07-10.38)
HPV67_b_	0.7 (0-2)	1 (0-2)	1 (0-2)	0.64* (0.33-1.13)	3.75 (1.94-6.56)	1.49 (0.77-2.61)	1 (0-3)	0	3.29 (1.70-5.75)	1.46 (0.76-2.56)
HPV26/69^b^	2(0-4)	1 (0-3)	3 (0-6)	2.62* (1.35-4.58)	6.79 (3.51-11.88)	3.43 (1.78-6.01)	0	2 (0-5)	6.60 (3.41-11.55)	5.96 (3.08-10.42)
HPV53/66^b^	5 (2-9)	12 (7-17)*	18 (12-24)^###^	9.83 (5.08-17.20)	12.80 (6.62-22.39)	17.05^#^ (8.82-29.84)	11 (5-17)	12 (6-18)	12.93 (6.68-22.62)	19.49 (10.08-34.10)
HPV68/70^b^	5 (2-9)	11 (5-15)	13 (8-19)^#^	7.91 (4.09-13.85)	9.01 (4.66-15.77)	11.30 (5.84-19.77)	15 (8-22)	12 (6-18)	10.36 (5.35-18.12)	12.18 (6.30-21.31)
HPV31/33/35^a^	8 (4-13)	14 (8-19)	10 (5-15)	6.87 (3.55-12.03)	7.52 (3.89-13.15)	8.85 (4.58-15.49)	13 (6-20)	6 (1-11)	6.34 (3.28-11.10)	7.77 (4.02-13.59)
										
**Low Risk**										
HPV6/11/42/44^c, d^	8 (4-13)	14 (8-19)	8 (4-13)	5.30 (2.74-9.27)	7.97 (4.12-13.95)	8.76 (4.53-15.32)	20 (12-28)^§^	8 (3-13)	7.59 (3.93-13.29)	8.41 (4.35-14.72)
HPV54/40^d^	3(0-5)	3 (0-5)	3 (0-6)	5.89 (3.04-10.31)	8.49 (4.39-14.85)	8.01 (4.14-14.02)	4 (0-8)	1 (0-3)	6.67 (3.45-11.68)	11.11 (5.74-19.44)
HPV13/32^d^	0.7 (0-2)	1 (0-2)	3 (0-5)	2.24 (1.16-3.92)	4.59 (2.37-8.03)	3.71 (1.92-6.50)	1 (0-3)	3 (0-6)	2.20 (1.14-3.85)	5.14 (2.66-8.99)
HPV62/72^d^	0.7 (0-2)	1 (0-2)	1 (0-3)	1.60 (0.83-2.80)	2.91 (1.51-5.10)	5.18^#^ (2.68-9.06)	0	0	1.09^§^ (0.56-1.91)	7.20 (3.72-12.60)
HPV2/57^d^	0	1 (0-2)	0	0	0.83 (0.43-1.46)	1.45^#^ (0.75-2.55)	1 (0-3)	0	0	1.43 (0.74-2.50)
HPV55^d^	0	0	0	0.96 (0.50-1.68)	1.66 (0.86-2.91)	0.36 (0.19-0.64)	0	0	0	0.71 (0.37-1.25)

^a ^high risk, ^b ^probably/possibly carcinogenic, ^c ^vaccine type, and ^d ^low-risk HPV type; * 0.01 > p > 0.05, **0.05 > p > 0.005, ***0.005 > p - HIV^+ ^before HAART initiation vs hiv^-^; ^# ^0.01 > p > 0.05, ^##^0.05 > p > 0.005, ^###^0.005 > p -HIV^+ ^after HAART initiation vs hiv^-^; ^+^0.01 > p > 0.05, ^++^0.05 > p > 0.005, ^+++^0.005 > p - before vs. after HAART initiation; ^§^0.01 > p > 0.05, ^§§^0.05 > p > 0.005, ^§§§^0.005 > p - before vs. after HAART initiation (with both follow-up).

For individual high-risk HPV types, incidence ranged from 0.64-9.83 cases/100 PY for HPV 67 and HPV53/66, respectively, among HIV-negatives; from 3.00-12.80 cases/100 PY for HPV 39 and HPV53/66, respectively, among the HIV-positives in the before HAART initiation period; and from 1.49-17.05 cases/100 PY for HPV 67 and HPV 53/66, respectively, among the HIV-positives in the after HAART initiation period (Table 2). Overall, the prevalence and incidence of individual HPV types tended to be higher among HIV-positive than HIV-negative individuals; however, the difference was statistically significant only for a few HPV types in the three groups (Table 2).

**Figure 1:**
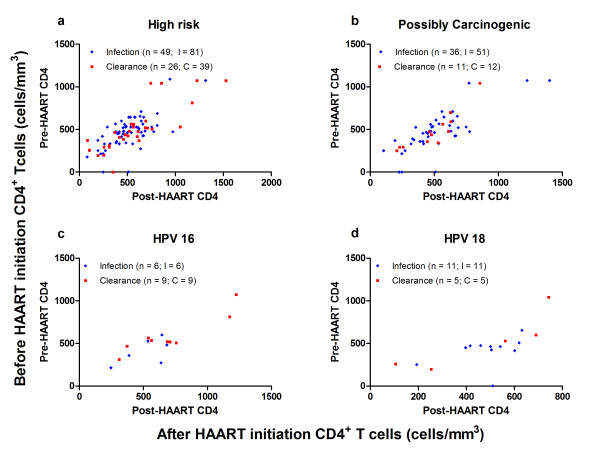
**Before (y-axis) and after (x-axis) HAART initiation CD4**^**+ **^**T-cell (CD4**^**+**^**) counts of type-specific new HPV events**. CD4^+ ^counts for all new infections (I) and clearance (C) for a) all high-risk HPV types (I:81 in 49 female adolescents, C:39 in 26 participants); b) possible carcinogenic types (I:51 in 36 participants, C:12 in 11 infections); c) HPV 16 (I:6 in 6 participants, C:9 in 9 participants); and d) HPV 18 (11 in 11 participants and 5 in 5 participants). For new infections, the before HAART initiation CD4^+ ^count was the mean from all pre-HAART HPV-negative visits, and the after HAART initiation CD4^+ ^count was the average value of the last post-HAART HPV-negative visit and the first HPV-positive visit. For clearance, the before HAART initiation CD4^+ ^count was the mean from all pre-HAART HPV-positive visits, and the after HAART initiation CD4^+ ^count was the average value of the last post-HAART HPV-positive visit and the first HPV-negative visit (at least two consecutive HPV-negative visits).

The absolute CD4^+ ^increased in a majority of the individuals in the subset analysis of individuals with both before and after HAART initiation follow-up periods. However, as shown in Figure 1(a-d), the absolute CD4^+ ^count or the change in CD4^+ ^as a result of immune reconstitution from HAART therapy did not affect infections and clearance patterns of individual HPV or combined carcinogenic HPV types. As a matter of fact, the prevalence and incidence rates of low-risk HPV types were similarly higher after HAART initiation than before HAART initiation. The incidence of high-risk HPV types 31/33/35, 53/66, 56, 58, 26/69, and 68/70 were all higher than HPV 16, while the incidence of types 53/66 and 68/70 were higher than HPV 18 in both before and after HAART initiation periods. Overall, the results suggest that HAART has no effect on high- or possible-carcinogenic HPV infections. There were more counts of infections than clearance in all HPV type groups except for the most potent carcinogenic HPV 16 strain (high-risk HPV types: 81 infections [in 49 women, n = 49] versus 39 clearance [n = 26]; possible carcinogenic HPV types: 51 infections [n = 36] versus 12 clearance [n = 11]; low-risk HPV types: 48 infections [n = 28] versus 8 clearance [n = 8]; HPV16: 6 infections [n = 6] versus 9 clearance [n = 9]; HPV18: 11 infections [n = 11] versus 5 clearance [n = 5]). Of note, the numbers for clearance were too small during the time period to stratify and compare either by different variables or by specific incidence cases.

## Discussion

The prevalence of HPV infection in our study subjects is high: 70% (262/373) at baseline and 70% (78/111) infected during follow-up, with a cumulative prevalence of 91% (340/373). This is as expected for a high-risk cohort of adolescents, judged by their self-reported sexual and other risk behaviors, and for HIV patients in the U.S. [30]. While clearance of HPV infection is frequent among HIV-positive adolescents on HAART, continued acquisition and persistence of HPV infections are common with and without HAART therapy, as previously reported [21,26,27,30]. HPV prevalence and incidence for most types tend to be slightly lower among HIV-negative than HIV-positive females in general (although this is not statistically significant in most cases--see Table 2 footnote); however, our data suggest that there is no immediate effect of HAART on HIV-infected adolescents, especially with regard to high-risk and vaccine-type HPV infection prevalence, persistence, or clearance. Additionally, there is no clear pattern of HPV infection or clearance with respect to immune reconstitution (based on CD4^+ ^T-cell counts) from HAART.

The present data corroborate previous findings that there is no immediate positive effect of HAART on high-risk HPV incidence and clearance, despite immune reconstitution through the increase of CD4^+ ^T-cells after treatment. On the contrary, we observed higher prevalence and incidence of possible carcinogenic and low-risk HPV types in the after HAART initiation period. Thus, prevention of HPV acquisition is important, especially in vulnerable populations such as sexually active adolescents. Recently, the FDA approved Merck's HPV vaccine, Gardasil [39], for use in girls and women aged 9-26, and the European Medicines Agency approved GlaxoSmithKline's HPV vaccine, Cervarix [40]. However, since there is no known therapy for HPV infections, measures to control acquisition or persistence of HPV infections (mainly other possible carcinogenic HPV types), especially in HIV/HPV co-infected females, would reduce the cervical cancer burden. Of note, high-risk types other than HPV 16 and 18 (targeted in both vaccines) occurred more frequently in the before and after HAART initiation periods. Thus, although the vaccines might be effective for particular 16 and 18 strains, innovative approaches are needed to examine cross-protection against other high-risk types that might be of epidemic concerns in HIV-positive populations both on and off HAART. Interestingly, the prevalence of HPV 16 was quite high, but the incidence was not that high compared to the other types and remained similar among HIV-negative participants and HIV-positive participants before and after HAART. Several studies have reported that the prevalence of HPV 16 was lower in HIV-positive women than in the general population and that other high-risk types are also present in these populations [41]. Most of these studies, however, are cross-sectional and lack prospective data, even for a shorter period like ours. While speculative, our data could indicate that HPV type 16 is more prevalent among the participants and their partners overall and thus may be transmitted during the earlier sexual encounters, whereas other HPV types may be in circulation in this population with higher incidence, but because they do not persist longer, their cumulative prevalence is lower.

The small size of our overall study sample limits our ability to perform analyses that would fully answer some relevant questions, such as the impact of HAART on type-specific clearance and persistence. Thus we present only counts of clearance rather than the rates. Additionally, the number of visits in which HPV data were obtained varied considerably from person to person, both during and before/after HAART initiation period, and may have introduced a systematic bias. However, the median number of visits for the two time periods was similar. The follow-up time periods might be shorter, especially to detect clearance with two consecutive negative visits, but they are relevant as they span the initiation of HAART and examine the immediate effect of HAART. The median follow-up time after HAART initiation in the study was 427.5 days. Most (70-90%) HPV infection in healthy women clears between 12-24 months, and the 13-month follow-up time may not be long enough to observe a significant difference and thus may lead to our result. Although we observed the reconstituted immunity (increase in CD4^+ ^count), it is possible that it may take a longer time to clear any infection, including HPV. The long-term period cannot be assessed from this study; however, it would be important in the future to study the long-term effect of HAART, especially in adolescents as they live to adulthood.

Further, it is possible that the use of cervical lavage samples, rather than cervical exfoliated cells samples, may have resulted in a slightly lower sensitivity for HPV detection, although we do not expect this to unduly affect our study results. Since HPV testing was performed semi-annually, it is possible that a proportion of infections of less than six-month duration were missed. Although this may affect the prevalence and incidence endpoints, more emphasis was placed on evaluation of HPV infections that persisted, since transient infections are less frequently associated with significant high-grade precancerous outcomes. The genotyping of HPV type was performed using a standard, validated method; however, some of the types were combined with a common probe for the analysis. This made it challenging to separate some of the HPV types, such as types 6 and 11, which are included in the quadrivalent HPV vaccine, from types 42 or 44. Additionally, persistence might be overestimated if the infections were different at consecutive visits but were detected by the generic probe, but clearance would be more conservative. However, high-risk and low-risk types still could be clustered for the analyses. In essence, participants of the study were high-risk adolescents, many of whom were likely recently infected with HPV. Thus, this will likely reduce the bias of long-term persistence in this population, specifically among those who were HPV-positive at baseline.

Besides HIV infection, the study participants were co-infected with other STIs [42], which may cumulatively or independently affect the immune system separately from HAART therapy. Although other factors, including age, sex, race, socio-economic status, smoking status, health-care, and host genetics, are associated with acquisition and clearance of HPV infection, these variables were not adjusted due to the small sample size. For instance, we did not observe any statistically significant associations (data not shown) with the incidence, prevalence, or clearance of type-specific HPV by baseline smoking status (40% of participants reported ever smoking). While these factors may be important, the present study focused only on the effect of HAART. All female adolescents who adhered to their medication were included and were censored either on the visit-date that they reported not taking their medication or at the end of the study. While validation of self-reports is not always reliable, this seems like an appropriate proxy for measuring their adherence, given the study design in this adolescent population. There are reports that Protease Inhibitor (PI) might inhibit the degradation of p53, which forms part of the mechanism of HPV persistence; however, we did not see any significant association of PI-based HAART (at baseline). Also, several participants changed their HAART regimen during follow-up, which makes further analysis of this association difficult, and the incidence of clearance post-HAART was limited.

## Conclusions

Although HAART increases the life expectancy of HIV patients, it did not show immediate effect on high-risk and vaccine-type HPV incidence, clearance, and persistence in our study. However, it is very important to monitor HIV-infected females receiving HAART for a longer period of time, particularly in regions of the world where cervical screening is not routinely available. Additional studies of other possible carcinogenic HPV types, along with the known HPV 16 and 18, which are highly prevalent in HIV-positive populations such as REACH, are also required for effective HPV prevention programs. More studies of the impact of HAART and immune restoration along with medication adherence on HPV infection and related outcomes are warranted to guide the development of interventions for such at-risk adolescents.

## List of abbreviations used

**DNA**: Deoxyribonucleic acid; **FDA**: Food Drug Administration; **HAART**: Highly Active Antiretroviral Therapy; **HIV**: Human Immunodeficiency Virus; **HPV**: Human Papillomavirus; **IQR**: Interquartile Range; **NHANES**: National Health and Nutrition Examination Survey; **PCR**: Polymerase Chain Reaction; **PY**: Person-years; **REACH**: Reaching for Excellence in Adolescent Care and Health; **STI**: Sexually Transmitted Infection

## Competing interests and Conflicts of interests

The authors declare that they have no competing interests.

## Authors' contributions

SSh conceived the idea, supervised the analysis and drafted the manuscript. SSu conducted the literature search and performed the analysis. JSS, LHB, CW, and MC-K assisted with the analysis and interpretation of the results. All authors read and approved the final manuscript.

## Pre-publication history

The pre-publication history for this paper can be accessed here:

http://www.biomedcentral.com/1471-2334/10/295/prepub
